# The national Fire and Fire Surrogate study: Effects of fuel treatments in the Western and Eastern United States after 20 years

**DOI:** 10.1002/eap.70003

**Published:** 2025-02-12

**Authors:** Alexis A. Bernal, Scott L. Stephens, Mac A. Callaham, Brandon M. Collins, Justin S. Crotteau, Matthew B. Dickinson, Donald L. Hagan, Rachelle Hedges, Sharon M. Hood, Todd F. Hutchinson, Melanie K. Taylor, T. Adam Coates

**Affiliations:** ^1^ Department of Environmental Science, Policy, and Management University of California, Berkeley Berkeley California USA; ^2^ USDA Forest Service, Southern Research Station Athens Georgia USA; ^3^ USDA Forest Service, Pacific Southwest Region Vallejo California USA; ^4^ USDA Forest Service, Rocky Mountain Research Station Missoula Montana USA; ^5^ USDA Forest Service, Northern Research Station Delaware Ohio USA; ^6^ Department of Forestry and Environmental Conservation Clemson University Clemson South Carolina USA; ^7^ Berkeley Forests University of California, Berkeley Berkeley California USA; ^8^ Odum School of Ecology University of Georgia Athens Georgia USA; ^9^ Department of Forest Resources and Environmental Conservation Virginia Tech Blacksburg Virginia USA

**Keywords:** fuels treatments, hardwood, mixed conifer, prescribed fire, restoration thinning, wildfire

## Abstract

The national Fire and Fire Surrogate (FFS) study was initiated more than two decades ago with the goal of evaluating the ecological impacts of mechanical treatments and prescribed fire in different ecosystems across the United States. Since then, 4 of the original 12 sites remain active in managing and monitoring the original FFS study which provides a unique opportunity to look at the long‐term effects of these treatments in different regions. These sites include California (Blodgett Forest Research Station), Montana (Lubrecht Experimental Forest), North Carolina (Green River Game Land), and Ohio (Ohio Hills). Although regions differed in ecosystem type (e.g., conifer‐ vs. hardwood‐dominated), the overall goals of the FFS study were to promote desirable, fire‐adapted species, reduce fire hazard, and improve understory diversity. Our study uses multivariate techniques to compare how these desired outcomes were maintained over the last 20 years and discusses whether we would modify the original treatments given what we know now. Our findings indicate that mechanical treatments and prescribed fire can promote desired tree species, mitigate potential fire behavior by reducing fuels and retaining larger‐sized trees, decrease tree mortality, and stimulate regeneration—effects that are still apparent even after 20 years. However, we also found that maintaining desired outcomes was regionally specific with western sites (California and Montana) showing more desirable characteristics under mechanical treatments, while the eastern sites (North Carolina and Ohio) showed more desirable characteristics after prescribed burning. The beneficial effects of treatment were also more apparent in the long term when sites followed up with repeated treatments, which can be adapted to meet new objectives and conditions. These findings highlight the FFS study as an invaluable resource for research and provide evidence for meeting long‐term restoration goals if treatments can be adapted to ecosystem type, be maintained by repeated treatments, and accommodate new goals by adapting treatments to changing conditions.

## INTRODUCTION

Mechanical and prescribed fire treatments are implemented to reduce fire risk and restore resiliency in forests with historically frequent fire regimes of low to moderate severity (Alexander et al., 2021; Hagmann et al., 2021; Nowacki & Abrams, 2008; Prichard et al., 2021). While these treatments can vary widely, objectives may or may not be mutually exclusive (Davis et al., 2024; Stephens et al., 2020). For example, mechanical treatments can mitigate fire behavior by reducing tree density, retaining larger‐sized trees, promoting fire‐resistant species, and reducing crown fire potential (Agee & Skinner, 2005; Brodie et al., 2024). The residual forest structure and composition following these treatments can also promote resiliency to disturbances and climate change (Bernal et al., 2022; North, Tompkins, et al., 2021). However, mechanical treatment alone does not replicate all the ecological processes resulting from fire, which may limit the function and integrity of frequent‐fire forests following disturbances. Conversely, prescribed fire can reintroduce and maintain important ecological processes while also reducing surface fuels. However, restoration goals may not be achieved with prescribed fire alone since operational conditions under which fire can be implemented often limit fire behavior, producing insufficient changes to stand structure or composition (Arthur et al., 2015; North, York, et al., 2021; Stephens et al., 2020; Waldrop et al., 2016; Zald et al., 2022). Therefore, balancing multiple objectives may necessitate a more integrated approach where some combination of mechanical treatments and prescribed fire are applied (Brose et al., 2013; Schwilk et al., 2009).

**FIGURE 1:**
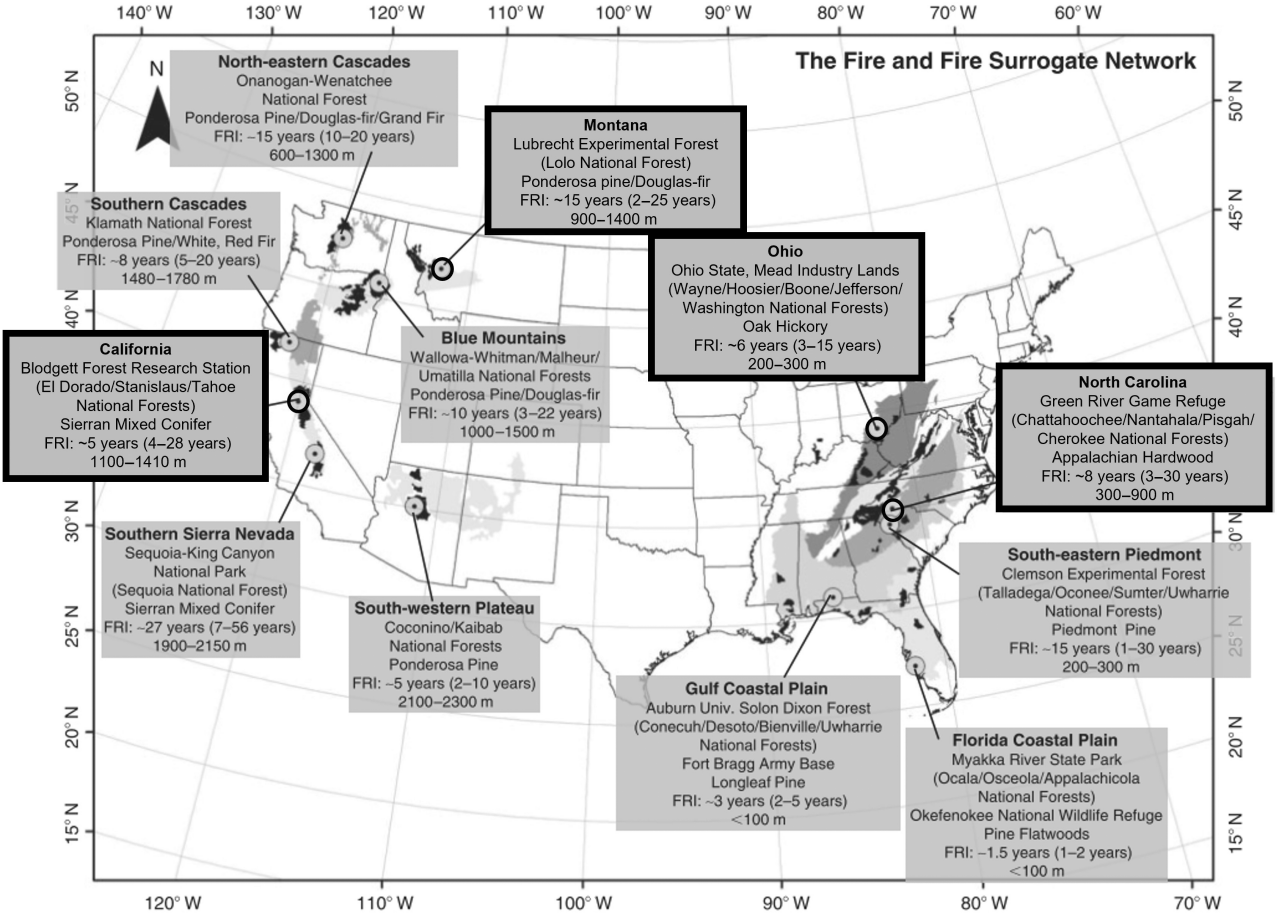
Name, location, forest type, fire return interval (FRI), and elevation (m) for each of the 12 Fire and Fire Surrogate (FFS) sites included in the initial treatments (adapted from Schwilk et al., 2009). The four active sites outlined in black include (from left to right of map) California (Blodgett Forest Research Station), Montana (Lubrecht Experimental Forest), Ohio (Ohio Hills), and North Carolina (Green River Game Land).

Over the last 20 years, efforts to mitigate fire behavior and enhance resilient forest characteristics included reducing fuels and tree densities while promoting large trees of fire‐adapted species. However, disparate studies evaluating the impacts of mechanical and prescribed fire treatments have made it difficult to characterize generalizable trends in forest conditions and treatment efficacy over time. The national Fire and Fire Surrogate (FFS) study is an invaluable endeavor to reconcile these discrepancies and provides a more comprehensive assessment of how commonly used fuel treatments achieve multiple objectives. Initiated in 2000, the FFS is a nationwide, collaborative effort to compare the ecological consequences of mechanical treatments and prescribed fire (McIver et al., 2009). A unique aspect of this study is the large‐scale network of experimental sites involved (12 sites across the United States) and the different ecosystems that were manipulated, ranging from seasonally dry pine forests in the West to hardwood forests in the East. Although national funding for the FFS network formally ended in 2009, four research sites have continued to monitor the effects of the original treatments, and in three instances, have applied follow‐up treatments to maintain or enhance the desired outcomes from the original treatments. These sites include Blodgett Forest Research Station, California; Lubrecht Experimental Forest, Montana; Green River Game Land, North Carolina; and Ohio Hills, Ohio (Figure 1). Although treatment prescriptions for each site varied to accommodate site‐specific ecology, desirable outcomes from the FFS generally included reducing fuels and fire hazard, promoting larger trees and preferred fire‐adapted overstory species, as well stimulating desirable tree regeneration and diverse understory communities (McIver et al., 2009).

While the ecological effects of mechanical and prescribed fire treatments have been widely studied since the FFS was first implemented, there is no other comparative study that encompasses such a wide range of ecosystems and does so with the longitudinal breadth encompassed by these four sites. Having two sites in the Eastern United States and two sites in the Western United States allows us to compare and contrast approaches to fuel and restoration treatments in frequent, low‐severity fire regimes in conifer and hardwood‐dominated forests. Although initial results from the 12 original sites indicated that a combination of mechanical treatments and prescribed fire maximized most ecological benefits, this finding was based on data taken 2–5 years after treatments were implemented (McIver et al., 2013; Schwilk et al., 2009; Stephens et al., 2009). Given the long‐term monitoring of these four sites, our objectives were to (1) summarize the long‐term (~20‐years post‐treatment) changes in overstory and understory composition, fuels and potential fire behavior, (2) describe which desired outcomes (if any) differentiate treatments over time, and (3) explore how treatments could be modified given what we learned about how stands have changed following treatments for over 20 years.

## METHODS

### Study sites

The four study sites that have continued monitoring the effects of fuel and restoration treatments from the FFS span a longitudinal gradient across the United States (Figure 1) and encompass state and federal jurisdictions. From here, sites will be referenced in the text and figures according to their geographic location: California (Blodgett Forest Research Station), Montana (Lubrecht Experimental Forest), North Carolina (Green River Game Land), and Ohio (Ohio Hills). While all sites are within regions that are considered to have relatively frequent historical fire regimes (~5–15‐year intervals) (Heyerdahl et al., 2008; Lafon et al., 2017; Schwilk et al., 2009; Stephens & Collins, 2004), species composition varies across a range of fire‐adapted species. The California site was historically dominated by pines (*Pinus* spp.), Montana was dominated by ponderosa pine (*P. ponderosa* var. *ponderosa*), while North Carolina and Ohio were dominated by oaks (*Quercus* spp.). However, changes in disturbance regimes including removal of indigenous burning, fire suppression, grazing, and logging practices have dramatically transformed these forests (Hagmann et al., 2021; Nowacki & Abrams, 2008). This includes increased dominance of shade‐tolerant fire‐sensitive species, increased tree densities, and higher densities of smaller‐sized trees, as well as higher fuel loads (Arthur et al., 2021; Hood et al., 2021; Knapp et al., 2017; Lydersen et al., 2013; North, Tompkins, et al., 2021; Stephens et al., 2009)—all of which render these forests highly vulnerable to high‐severity disturbances (Stephens et al., 2018). Although the original intent of the FFS focused primarily on immediate wildfire mitigation, particularly among the western sites, reducing the vulnerability of these forests by using treatments that enhance resistance may produce additional benefits that maintain ecological integrity and, thus, protect ecosystem services in the long term.

### Experimental design and treatments

Each site was established in 2000, where four treatments were implemented including an untreated control (Control), prescribed fire only (Fire), mechanical treatment only (Mech), and mechanical treatment followed by prescribed fire (Mech + Fire). At all sites, each treatment was replicated in three blocks (except at the California site, which used a completely randomized design) for a total of 12 experimental units. Each unit ranged from 9 to 29 ha in size, with mechanical treatments applied between 2000 and 2002 and the first‐entry prescribed fire applied between 2001 and 2003. Although the details of each treatment are site‐specific (Table 1), the primary goal of the FFS was to modify stand structure and composition so that 80% of the dominant and codominant trees would survive a wildfire under 80th percentile weather conditions (McIver et al., 2013). In general, thinning treatments focused on removing lower and mid‐story canopy trees, with some of the sites (California, Montana, and Ohio) giving preference to retaining site‐specific species that were dominant under an intact fire regime (e.g., pines, western larch [*Larix occidentalis* Nutt.], and oaks). California and North Carolina conducted two rounds of mechanical treatments, with California adding mastication as an additional fuel treatment post‐harvest. Prescribed fire was implemented at least once at each site but ranged from 1 to 4 fires, which produced variable fire behavior across sites and time. In 2019, the California site included an additional salvage harvest in the Mech + Fire units following prescribed fire conducted in the previous year (Table 1).

**TABLE 1:** Summary of site‐specific applications of treatments including Mech (mechanical treatment only), Fire (fire only), and Mech + Fire (mechanical treatment followed by prescribed fire).

Site	Mech		Fire		Mech + Fire	
	Year	Overview	Year	Overview	Year	Overview
California^a^	2001	Mechanically thinned from below; retained 28–34 m^b^ ha^−1^ BA and even species mix for retention; masticated 90% of trees <25 cm dbh	2002	Broadcast burned (strip head‐fire, fall)	2001	Mechanically thinned from below; retained 28–34 m^b^ ha^−1^ BA and even species mix for retention; masticated 90% of trees <25 cm dbh
	2017	Mastication	2009	Broadcast burned (strip head‐fire, fall)	2002	Broadcast burned (backing fire, fall)
	2019	Mechanically thinned from below	2017	Broadcast burned (strip head‐fire, fall)	2017	Mastication
					2018	Broadcast burned (backing fire, fall)
					2019	Salvage harvest
Montana^b^	2001	Mechanically thinned from below, retained ~11 m^b^ ha^−1^ BA; pines and western larch ≥40 cm dbh preferred for retention	2002	Broadcast burned (strip head‐fire, spring)	2001	Mechanically thinned from below, retained ~11 m^b^ ha^−1^ BA; pines and western larch preferred ≥40 cm dbh preferred for retention
					2002	Broadcast burned (strip head‐fire, spring)
Ohio^c^	2000–2001	Mechanically thinned from below (<35 cm dbh); retained ~70% BA; oaks and hickory preferred for retention	2001	Broadcast burned	2001	Mechanically thinned from below (<35 cm dbh); retained ~70% BA; oaks and hickory preferred for retention; Broadcast burned
			2005	Broadcast burned	2005	Broadcast burned
			2010	Broadcast burned	2010	Broadcast burned
			2016	Broadcast burned	2016	Broadcast burned
North Carolina^d^	2002	Hand thinned from below (<10.2 cm dbh) regardless of species, and all shrubs regardless of size	2003	Broadcast burned (spot fire + helicopter ignition, winter)	2002	Hand thinned from below (<10.2 cm dbh) regardless of species, and all shrubs regardless of size
	2012	Hand thinned from below (<10.2 cm dbh) regardless of species, and all shrubs regardless of size	2006	Broadcast burned (spot fire + hand ignition, winter)	2003	Broadcast burned, winter
			2012	Broadcast burned (spot fire + hand ignition, winter)	2006	Broadcast burned, winter
			2015	Broadcast burned (spot fire + hand ignition, winter)	2012	Broadcast burned, winter
					2015	Broadcast burned, winter

*Note*: Presented are years when treatments were conducted and general overviews of the prescription that was applied including type of thinning, basal area (BA) retention following thinning, dbh limits, preferential species retained, and type and season of prescribed fire implemented. Site‐specific papers detailing prescriptions are indicated. See Appendix S1: Table S1 for definitions of mechanical and prescribed fire operations.

^a^ Stephens and Moghaddas (2005).

^b^ Fiedler et al. (2010).

^c^ Hutchinson et al. (2024).

^d^ Waldrop et al. (2016).

All sites used a systematic grid at 50‐ or 60‐m spacing to randomly select plot locations (Table 2). Montana and Ohio used 20 × 50 m (0.10 ha) modified Whitaker plots (Keeley & Fotheringham, 2019; Metlen & Fiedler, 2006) to collect data on overstory, understory, and fuel loads, while North Carolina used all grid points to measure fuels and a subset of 10 grid points in each treatment unit to establish modified Whitaker plots to measure overstory and understory, and California used 11.3‐m radius (0.04 ha) circular plots to measure all metrics (Stephens & Moghaddas, 2005). Pretreatment data were collected between 2000 and 2001 and will collectively be referred to as Pre‐treatment. While multiple posttreatment measurements were taken during different years across sites, we will report the last (i.e., most current; 17–22 years posttreatment) measurements taken at each site, which will be referred to as Post‐20.

**TABLE 2:** Summary of data collection across sites including years when Pre‐treatment (Pre) and Post‐20 (Post) were taken.

Site	Pre	Post	Layout	Seedling, sapling, overstory	Understory	Fuels	Sample size/unit
California	2001	2020	60‐m grid	0.004, 0.04‐ha circular	0.004‐ha subplot	Two transects (11.3 m)	60 plots (*n* = 240)
Montana	2000	2020 and 2022	50‐m grid	0.0001, 0.01, 0.10‐ha modified Whitaker	0.0001‐ha subplot	Two transects (15.2 m)	10 modified Whitaker; 36 transects (*n* = 120; *n* = 432)
Ohio	2000	2021	50‐m grid	0.002, 0.03, 0.10‐ha modified Whitaker	0.0012‐ha subplot	Two transects (20 m)	30 plots (*n* = 120)
North Carolina	2001	2018	50‐m grid	0.0001, 0.01‐ha modified Whitaker	0.01‐ha subplot	Three transects (15.2 m)	10 modified Whitaker; 40 transects (*n* = 120; *n* = 480)

*Note*: Columns from left to right, describe the layout of plots that were randomly selected, size (in hectares) and type of plot used to measure live seedling, live sapling, and mature tree metrics (overstory), size (in hectares) of plot used to measure understory metrics, number of planar intersect transects (length in meters; Brown, 1974) and methods used to measure fuels at each plot, and sample size for each unit (total of all four treatments for each site).

### Vegetation measurements—Overstory and understory

Although plot measurements varied at each site (Table 2), all mature trees within plots at each site were tagged and recorded for status (i.e., alive and dead), species, and dbh (at height of 1.37 m). Although tree regeneration (i.e., seedlings and saplings) is typically considered an understory component, we included it in the overstory analysis to demonstrate the potential for future overstory recruitment of preferred or non‐preferred tree species as well as fire hazard over time. In California, a minimum dbh of 15 cm was used to inventory mature trees within the entire plot (0.04 ha), while live seedlings (<dbh in height) and saplings (≥dbh in height and <15 cm dbh) were tallied by species in either a subplot (0.004 ha) or the entire plot depending on size. Montana, Ohio, and North Carolina had a minimum dbh of 10 cm to inventory mature trees. North Carolina measured mature trees in half of the 0.10‐ha subplots, while Montana and Ohio measured mature trees in all 0.10‐ha plots. At these sites, live saplings (≥dbh in height and <10 cm dbh) and seedlings (<dbh in height) were tallied by species in subplots of 0.01 and 0.0001 ha, respectively. Ocular estimates of percent cover of grasses, forbs, and shrubs by species were recorded in subplots at all sites but varied in terms of size in California (0.004 ha), Montana (0.0001 ha), Ohio (0.0012 ha), and North Carolina (0.01 ha for shrubs >1.37 m and 0.0001 ha for herbaceous vegetation and shrubs <1.37 m).

### Fuels measurements and potential fire behavior

Each site used a modification of planar intersect fuels protocols (Brown, 1974), establishing 2–3 transects (11.3–20 m in length) at each plot (Table 2). On each transect, duff and litter depths (in centimeters) were measured, as well as tallies of 1‐h (woody material < 0.64 cm diameter), 10‐h (0.64 cm ≤ diameter < 2.54 cm), 100‐h (2.54 cm ≤ diameter < 7.62 cm), and 1000‐h + (diameter ≥7.62 cm) timelag classes. For California and Montana, fire behavior predictions were generated using the Fire and Fuels Extension of Forest Vegetation Simulator (FFE‐FVS) (Rebain, 2015) using plot scale, mature tree measurements (i.e., canopy fuels) and surface fuels data. In FFE‐FVS, surface fuels were represented by either Scott and Burgan's (2005) modified 40 fuel models (California) or Anderson's (1982) 13 original fuel models (Montana). The default algorithm to assign fuel models and weights was used in Montana, while California used a more extensive model selection and weighting process (see Stephens et al., 2024 in this Special Feature). Potential fire behavior was assessed using two outputs generated from FFE‐FVS including probability of torching (P‐torch) and potential mortality (P‐mort) which is expressed as a percentage of basal area killed (Rebain, 2015). For Ohio and North Carolina, surface fuel models, which are a required input for FFE‐FVS and many other US‐based fire behavior models, do not represent hardwood ecosystems in this region well and are less reliable than those offered in conifer‐dominated forests in the west (Phillips et al., 2006). Therefore, fire behavior predictions were not generated for these sites.

### Treatment outcome metrics

Measurements were aggregated into three groups: overstory, understory, and fuels/fire behavior at the plot level for each site. To assess changes in overstory, understory composition, fuels, and fire behavior across treatments and time, we used plot averages for all measurements taken Pre‐treatment and Post‐20. For overstory structure, common metrics to describe mature overstory characteristics were calculated, including live and dead tree density (trees per hectare), basal area (in square meters per hectare), percentage of basal area occupied by site‐specific preferred species (e.g., pines, western larch, oaks, and hickory), and live quadratic mean diameter (QMD; in centimeters). Potential recruitment into the overstory was calculated as live seedling and sapling density (per hectare) of preferred (same species above) and non‐preferred species (species other than preferred) separately. Due to the limitations of hand‐thinning in North Carolina, a substantial amount of mid‐story shrubs resprouted following mechanical treatments. Therefore, we also included percent cover of mid‐story shrubs for this site as an overstory metric.

For understory composition, percent cover by species was aggregated to the genus level due to inconsistencies in data collection, as well as aggregated by lifeform (graminoid, forb, shrub, tree, and vine) and non‐native cover (Montana only). From genus percent cover, we also calculated three metrics to describe understory communities including diversity index (Shannon & Weaver, 1949), richness (total count of genera present), and Pielou's index of evenness (Pielou, 1966). We then aggregated fuel loads (in megagrams per hectare) for fine (1‐, 10‐, and 100‐h) fuels, coarse woody debris (CWD; 1000‐h+) and ground (i.e., litter and duff) fuels for California, Montana, and North Carolina, as well as plot‐level fire behavior outputs P‐torch and P‐mort for California and Montana. Due to inconsistent data collection Pre‐treatment, fuel data from Ohio are excluded from this dataset. Changes (∆) across treatments over time were then calculated as the difference between Post‐20 measurements and Pre‐treatment measurements (Post‐20 − Pre‐treatment) (Table 3).

**TABLE 3:** Summary of average changes (Post‐20 − Pre‐treatment, ∆) in overstory, understory, and fuel/fire behavior metrics across Control (C), Fire (F), Mech (M), and Mech + Fire (M + F) treatments at each site.

	California				Montana				Ohio				North Carolina			
	C	F	M	M + F	C	F	M	M + F	C	F	M	M + F	C	F	M	M + F
Overstory																
∆live TPH	−60	−226	−281	−331	−67	−127	−188	−235	2	−192	−18	−239	−7	−148	−91	−349
∆dead TPH	27	17	−10	1	23	40	−3	9	−19	−15	−33	−36	−41	−6	−2	−12
∆live BA	13.47	2.13	−6.83	−18.28	−3.61	−3.78	−3.31	−7.95	5.38	−5.68	−2.47	−8.53	−1.74	−6.40	−1.59	−11.87
∆dead BA	4.08	1.25	−1.20	2.94	−1.07	−0.27	−0.29	−0.10	−0.90	−0.18	−1.78	−1.12	−5.25	−1.77	−1.45	−3.16
∆% BA preferred	−1.83	0.89	1.73	6.33	−62.59	−42.95	−23.78	−10.26	−3.69	−0.09	−6.39	7.82	−0.15	0.01	0.58	−7.60
∆live QMD	6.34	20.33	22.03	31.18	−0.19	1.10	7.63	10.53	2.92	10.09	−0.74	13.60	−0.44	0.20	1.31	2.11
∆Preferred seedlings	3720	−1210	5097	−1861	−431	−595	458	592	4756	9622	11,148	5789	167	1850	733	7133
∆NP seedlings	−449	2898	1798	−756	1345	172	1300	100	11,158	8782	1769	8925	NA	NA	NA	NA
∆Preferred saplings	−16	−147	−21	0	485	−115	472	30	1	2039	326	1948	82	202	41	2139
∆NP saplings	−128	−118	−403	−31	1268	525	1208	206	−6	4235	645	4333	NA	NA	NA	NA
∆Mid‐story shrubs	NA	NA	NA	NA	NA	NA	NA	NA	NA	NA	NA	NA	7.2	−17.87	−52.27	−22.32
Understory																
∆Graminoids	−1.75	−1.98	−2.51	−3.49	5.91	6.44	5.86	5.21	−0.20	1.91	−0.41	3.04	0.03	1.76	0.02	2.85
∆Forbs	−10.09	−7.60	−12.61	−11.	5.71	6.11	4.46	2.73	−0.85	6.18	0.05	8.16	−0.52	2.31	0.25	4.28
∆Shrubs	−5.82	−1.17	−11.20	1.31	8.13	8.14	6.27	4.19	−3.40	5.01	−4.08	6.88	−1.89	0.87	5.97	1.79
∆Trees	0.13	−5.75	−4.60	−1.48	NA	NA	NA	NA	1.49	26.21	3.92	24.23	NA	NA	NA	NA
∆Vines	NA	NA	NA	NA	NA	NA	NA	NA	NA	NA	NA	NA	−0.24	0.44	0.37	0.67
∆Non‐natives	NA	NA	NA	NA	−0.01	−0.01	−0.17	0.20	NA	NA	NA	NA	NA	NA	NA	NA
∆Diversity	−1.21	−1.18	−1.37	−1.41	0.06	0.11	−0.09	−0.14	0.12	0.38	0.26	0.51	−0.12	0.09	0.01	0.39
∆Richness	−4.82	−4.47	−6.24	−5.52	−6.43	−4.37	−5.67	−4.50	−2.13	8.87	1.37	11.73	−2.23	−4.30	−0.24	9.10
∆Evenness	−0.33	−0.32	−0.36	−0.39	−0.01	0.01	−0.05	−0.05	0.06	−0.09	0.06	−0.09	0.01	0.01	−0.01	0.08
Fuels/fire behavior																
∆Fine	0.77	−4.49	3.30	−6.18	9.17	6.90	1.88	3.09	NA	NA	NA	NA	1.39	3.56	5.05	4.85
∆CWD	−1.86	−10.23	−12.74	−16.39	27.39	24.50	0.54	4.04	NA	NA	NA	NA	3.60	10.39	1.63	23.92
∆Ground	42.23	−35.65	23.86	−52.74	19.20	13.45	17.87	10.57	NA	NA	NA	NA	2.79	−7.26	0.05	−12.23
∆P‐torch	24.98	−14.54	−23.33	−35.37	0.17	0.03	−11.00	−11.00	NA	NA	NA	NA	NA	NA	NA	NA
∆P‐mort	4.58	−31.47	−27.88	−36.91	0.46	0.28	0.09	−0.04	NA	NA	NA	NA	NA	NA	NA	NA

*Note*: Overstory metrics include changes in live and dead tree density (TPH; trees per hectare), basal area (BA; in square meters per hectare), percent BA of preferred species, live quadratic mean diameter (QMD, in centimeters), seedlings and saplings per hectare of preferred and non‐preferred (NP) species, and percent cover of mid‐story shrubs. Understory metrics include changes in life form (graminoids, shrubs, trees, and vines) percent covers, non‐native percent cover, and genera diversity, richness, and evenness. Fuels and fire behavior metrics include changes in fine fuels, coarse woody debris (CWD), ground fuels, probability of torching (P‐torch), and potential mortality (P‐mort). NA values indicate that data were not included in the canonical discriminant analysis (CDA).

### Statistical methods

To test whether treatments differ over time, absolute overstory, understory, and fuels/fire behavior metrics were tested using a multi‐response permutation procedure (MRPP) from the vegan package (Oksanen et al., 2022) in R (R Core Team, 2022), which is a nonparametric alternative to a multivariate analysis of variance. To do this, we had to aggregate plot data to the replicate level (*n* = 12 for each site) for Pre‐treatment and Post‐20 to avoid pseudo‐replication. Using ∆overstory metrics, ∆understory metrics, and ∆fuels and fire behavior, we conducted a canonical discriminant analysis (CDA) at the plot level (*n* = 120–240) to visualize and compare whether desired outcomes differentiated treatments 20 years after the initial treatments were implemented. CDA is a multivariate technique used to separate populations given a set of variables that are condensed down into a lower dimensional space (canonical axes). Unlike other multivariate techniques such as principal components analysis or nonmetric multidimensional scaling, CDA highlights which multivariate attributes segregate preestablished groups (e.g., treatments) which can then be used to make inferences about ecological relationships across treatments over time (sensu Crotteau et al., 2020). We conducted this analysis using the candisc package (Friendly & Fox, 2021) in R for each site using treatment (Control, Mech, Fire, and Mech + Fire) as our predefined groups and each measurement within the corresponding ∆overstory metrics, ∆understory metrics, and ∆fuels and fire behavior as our canonical variables. Wilk's Lambda values were calculated from the eigenvalues of each CDA and converted to *F*‐statistics to determine whether canonical correlations were significant.

Although individual study sites differed in how they measured fuels, fire behavior, overstory structure, and understory composition, the general principles for assessing treatment outcomes remained the same. Treatment outcomes were assessed by determining how treatment outcomes changed within a particular site over time and whether those outcomes differed across treatments. Outcomes included management objectives that were specific to each site including reducing fuels and fire hazard, promoting larger trees and preferred species composition, as well as stimulating diverse understory communities. Whether these desired outcomes persisted over the last 20 years is a function of site‐specific conditions that vary across our research areas, which can be difficult to synthesize and interpret if all sites were analyzed in a single cohesive model. However, evaluating desired outcomes on a site‐by‐site basis allowed us to overcome this limitation despite differing methodologies and conditions among sites. Although generalizable patterns across sites are discussed, a single comparative analysis including all sites was not performed. To learn more about the individual sites, their management history, experimental design, and calculations, see the site‐specific papers included in this Special Feature (e.g., Hood et al., 2024; Hutchinson et al., 2024; Stephens et al., 2024).

## RESULTS

### Overstory

**FIGURE 2:**
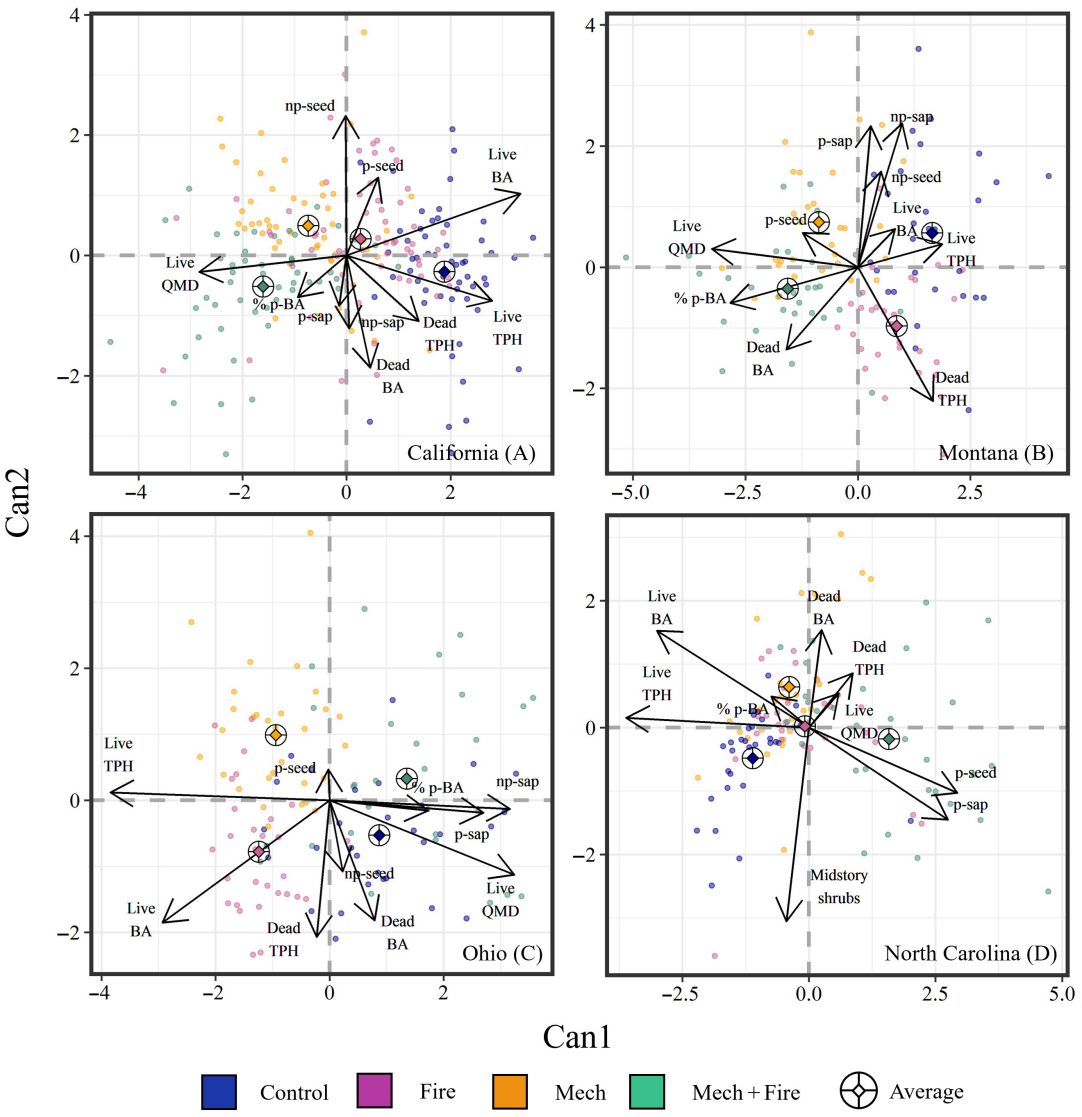
Canonical discriminant analysis (CDA) of changes (Post‐20 − Pre‐treatment) in overstory metrics in relation to treatments for (A) California, (B) Montana, (C) Ohio, and (D) North Carolina. Metrics include changes in percent basal area of preferred (p) species, live basal area (BA), live tree density (TPH), live quadratic mean diameter (QMD), seedling (seed) and sapling (sap) density of preferred species and non‐preferred (np) species, and mid‐story shrubs (North Carolina). Diamond within ringed cross indicates average score for a given treatment. Arrows represent correlation coefficients for a given metric.

All treatments had similar overstory within‐site characteristics prior to treatment with MRPP showing no difference in California (*p* = 0.65), Montana, (*p* = 0.99), Ohio (*p* = 0.80), and North Carolina (*p* = 0.45). After ~20‐years posttreatment, treatments were still differentiated by changes in overstory characteristics for California (*p* = 0.02), Montana (*p* = 0.01), Ohio (*p* = 0.01), and North Carolina (*p* < 0.01). CDA of ∆overstory for each site showed that a high percentage of variation in our datasets in California, Montana, Ohio, and North Carolina were explained by canonical axis 1 (Can1) and canonical axis 2 (Can2) combined (93%, 92%, 93%, and 92%, respectively; Appendix S1: Table S2). Can1 and Can2 were correlated with treatments, suggesting that desirable outcomes in mature overstory characteristics and regeneration could still be achieved even ~20 years after treatment in California, Montana, Ohio (*p* < 0.01 and *p* < 0.01 for Can1 and Can2, respectively), and North Carolina (*p* < 0.01 and *p* = 0.03, respectively). However, the desired outcomes that were associated with treatments varied by region. Desired outcomes such as larger trees, higher percent basal area of preferred tree species, and lower live basal area and tree density differentiated mechanical treatments (with or without fire) in the pine mixed‐conifer forests of California (Figure 2A) and Montana (Figure 2B). While these desired outcomes also separated treatments in the hardwood‐dominated forests of Ohio (Figure 2C) and North Carolina (Figure 2D), Mech + Fire appeared to be the most effective at retaining larger trees and preferred tree species while Mech did little to promote desired conditions. Both mechanical treatments (with or without fire) generally showed increases in seedling and sapling densities through ~20 years of treatment across all sites for both preferred and non‐preferred species, suggesting that recruitment of preferred species into the overstory would require adjusting future thinning intensities to accommodate shade tolerance of preferred species which may vary by region. Increased mortality also differentiated fire treatments (either thinned or unthinned) for all sites, which indicates that either direct or secondary mortality is a trade‐off to using prescribed fire.

### Understory

**FIGURE 3:**
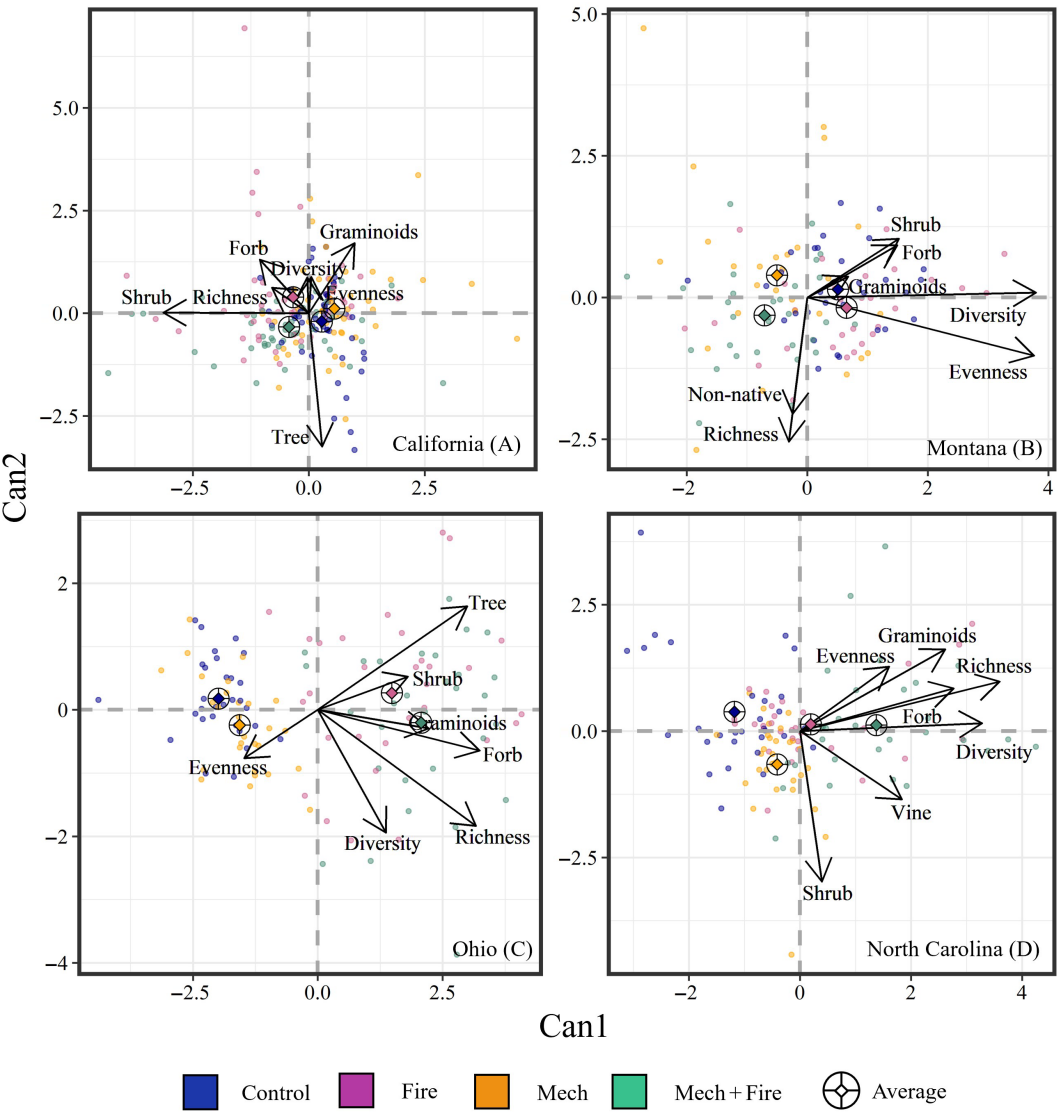
Canonical discriminant analysis (CDA) of changes (Post‐20 − Pre‐treatment) in understory metrics in relation to treatments for (A) California, (B) Montana, (C) Ohio, and (D) North Carolina. Metrics include changes in species richness, diversity, evenness, graminoid cover, forb cover, shrub cover, tree cover (California and Ohio), vine cover (North Carolina), and non‐native cover (Montana). Diamond within ringed cross indicates average score for a given treatment. Arrows represent correlation coefficients for a given metric.

Prior to treatment, all treatments had similar within‐site understory characteristics, with MRPP showing no difference across treatments in California (*p* = 0.99), Montana, (*p* = 0.16), Ohio (*p* = 0.37), and North Carolina (*p* = 0.43; Appendix S1: Table S2). After ~20 years since the first treatments were implemented, changes in understory characteristics remained differentiated in Ohio and North Carolina (*p* < 0.01) where fire was repeated, while treatments in California and Montana showed similar trajectories over time (*p* = 0.56 and *p* = 0.99, respectively). CDA of ∆understory for each site showed that a high percentage of variation in our datasets in California, Montana, Ohio, and North Carolina were explained by Can1 and Can2 combined (85%, 94%, 99%, and 96%, respectively). However, desired understory characteristics varied by treatment and region (Figure 3). In the hardwood‐dominated forests of Ohio (Figure 3C) and North Carolina (Figure 3D), greater cover of all life forms differentiated fire treatments (either thinned or unthinned; *p* < 0.01 and *p* = 0.04, respectively). While greater diversity metrics also separated fire treatments (either thinned or unthinned) in North Carolina (*p* < 0.01), any effect of fire treatments on diversity in Ohio largely disappeared ~20 years after treatment (*p* = 0.72). In the pine mixed‐conifer forests of Montana (Figure 3B), greater diversity metrics differentiated unthinned treatments (Fire and Control, *p* < 0.01), but any treatment differences in life form cover had largely disappeared ~20 years after treatment (*p* = 0.48). For California (Figure 3A), all treatments converged toward a *z* score of 0 indicating that changes in desired understory characteristics post‐treatment were not apparent ~20 years later.

### Fuels and potential fire behavior

**FIGURE 4:**
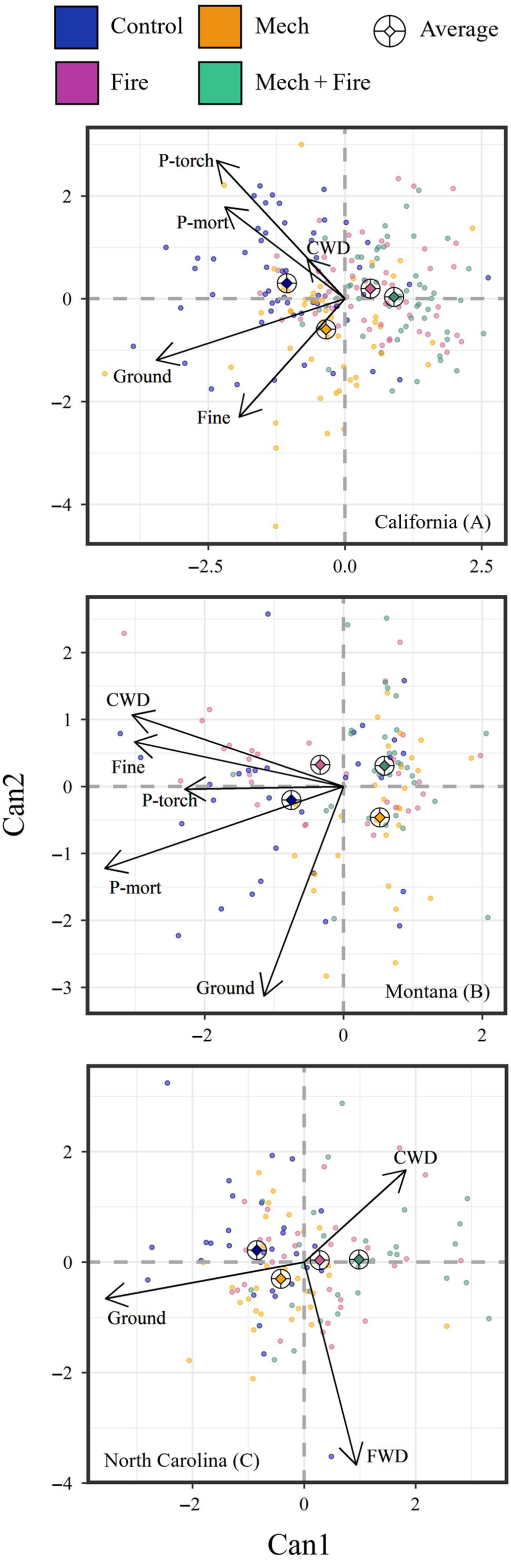
Canonical discriminant analysis (CDA) of changes (Post‐20 − Pre‐treatment) in fuels metrics in relation to treatments for (A) California, (B) Montana, and (C) North Carolina. Metrics include changes in fuel loads of ground (litter + duff), fine woody fuels (FWD; 1‐, 10‐, 100‐h), coarse woody debris (CWD; 1000‐h+), potential mortality (P‐mort; California and Montana), and probability of torching (P‐torch; California and Montana). Diamond within ringed cross indicates average score for a given treatment. Arrows represent correlation coefficients for a given metric.

Prior to treatment, all treatments had similar within‐site fuels (California, Montana, and North Carolina) and fire behavior (California and Montana only) with MRPP showing no difference across treatments in California (*p* = 0.99), Montana (*p* = 0.36), and North Carolina (*p* = 0.97; Appendix S1: Table S2). After ~20 years post‐treatment, treatments were still differentiated by changes in fuels and fire behavior in California (*p* < 0.01) and Montana (*p* = 0.04), but not in North Carolina (*p* = 0.22). CDA of ∆fuels and fire behavior showed that a high percentage of variation in our datasets in California, Montana, and North Carolina were explained by Can1 and Can2 combined (99%, 98%, and 99%, respectively). However, the types of fuels and fire behavior that differentiated treatments were regionally specific (Figure 4). For the pine‐dominated forests of California (Figure 4A) and Montana (Figure 4B), reductions in fire behavior metrics such as P‐mort and P‐torch differentiated mechanical treatments (with or without fire; *p* < 0.01). While fire treatments (either thinned or unthinned) were also differentiated by lower ground and fine woody fuels in California (*p* < 0.01), mechanical treatments (with or without fire) in Montana were differentiated by lower CWD (*p* < 0.01). In the hardwood‐dominated forests of North Carolina (Figure 4C), reduced ground fuels differentiated fire treatments (either thinned or unthinned) while changes in fine and coarse woody fuels were no longer different ~20 years after treatment (*p* = 0.11).

## DISCUSSION

The FFS provides evidence that managers can use a variety of tools to meet management goals and that, depending on region, fuels reduction and restoration do not have to be conflicting objectives (Stephens et al., 2020). We found that Mech and Mech + Fire can maintain low fuel loads, tree densities, and basal area in pine‐dominated forests—important structures for mitigating fire behavior and dominant characteristics of resilient landscapes. By retaining large trees of fire‐adapted species with Mech and Mech + Fire, western sites (California and Montana) not only lowered potential fire behavior, but also promoted conditions somewhat similar to what was present under frequent, low‐moderate severity fire regimes. However, we also found that the optimal treatment to achieve fuel hazard reduction and restoration may vary based on ecosystem (i.e., pine‐dominated vs. hardwood‐dominated), which can create trade‐offs for desired outcomes. While Mech in the eastern sites (Ohio and North Carolina) likely lowered potential fire behavior by reducing fuels, doing so also promoted non‐desired species (e.g., mesophytes that hinder fire‐adapted species by limiting fire) and showed similar understory and fuel conditions relative to the Control which suggests that Mech treatments in these types of systems may not be sufficient for achieving long‐term goals of forest restoration. Therefore, accounting for context of place is crucial for restoring conditions that are adapted to these disturbances and, thus, enhance the ecological function and integrity of these ecosystems.

**FIGURE 5:**
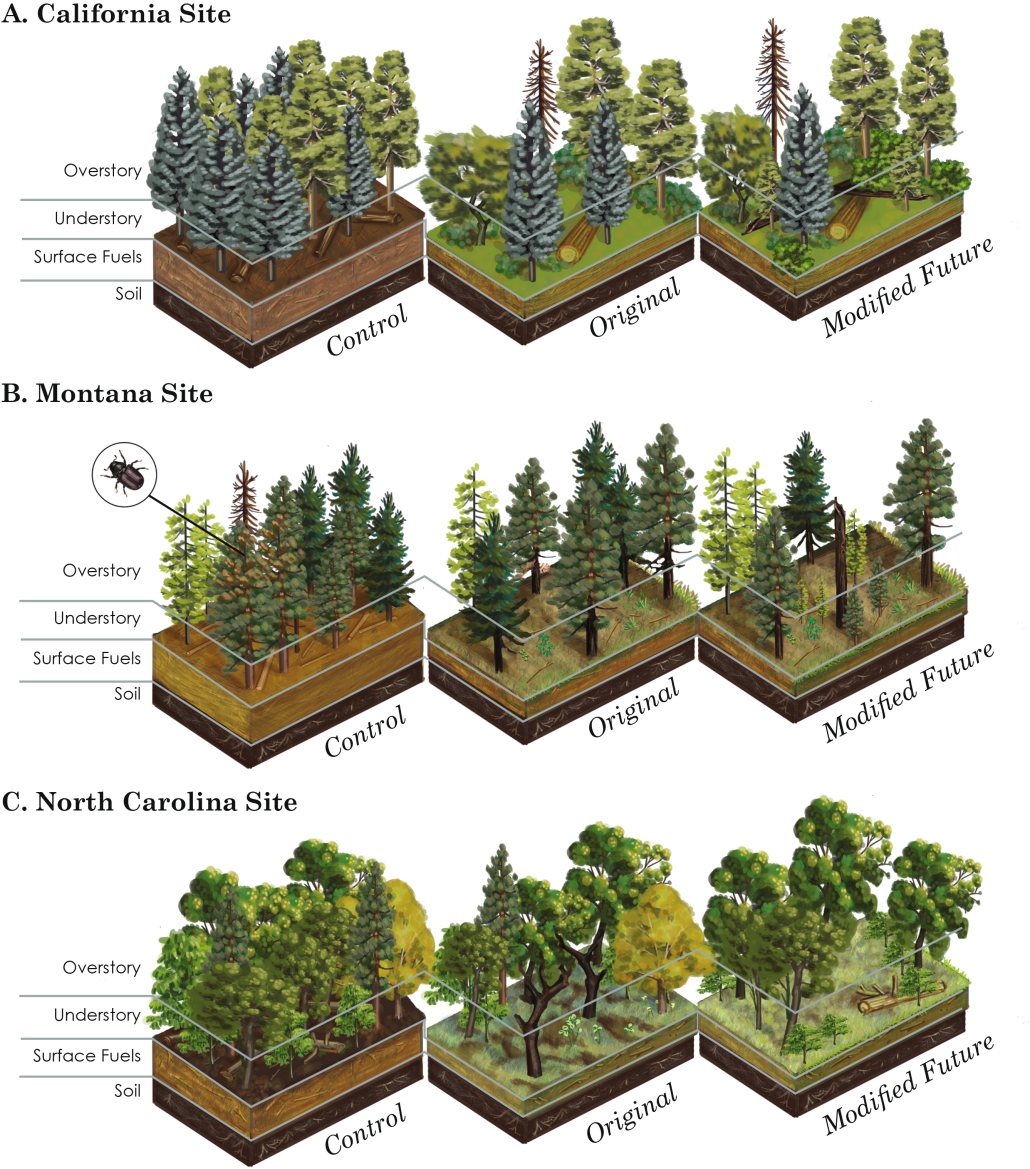
Illustration of overstory, understory, surface fuels, and soil characteristics prior to the FFS (Control) in (A) California, (B) Montana, and (C) North Carolina, our original prescription to change these characteristics so we could meet fuel hazard reduction goals (Original), and how we would change the original prescription given what we know about the development of these prescriptions the last 20 years (Modified future). Illustration credit: Allison Fitzmorris.

However, we would be remiss if we did not critically evaluate the original prescriptions that were implemented at each of these sites. Although various treatments continue to provide ecological benefits 20 years after treatment implementation, our observations of forest change over time have helped us identify modifications to the original prescriptions that may better accommodate changing environmental conditions, management objectives, and best practices for a particular forest type. We present a conceptual diagram (Figure 5) outlining the fuel hazard and forest structure conditions of California, Montana, and North Carolina prior to the FFS (Control), an overview of the original objectives we wanted to achieve (Original), and what we would change about the original prescription given what we know 20 years later (Modified Future). Due to limited information available for Ohio, this site is omitted from the conceptual diagram and discussion.

### California site

Fire was once a common process in the mixed‐conifer forests at this site. Between 1750 and 1900, median composite fire intervals at the 9–15‐ha spatial scale were 4.7 years with a fire interval range of 4–28 years (Stephens & Collins, 2004). Prior to treatments occurring at the California site, stands were composed of high tree densities and surface fuels, as well as less fire‐resistant species, such as firs and incense‐cedar (*Calocedrus decurrens* (Torr.) Florin), with little understory vegetation and regeneration (Figure 5A; Control). Leaving stands in this state rendered them highly vulnerable to disturbances such as drought, wildfire, and insect outbreaks—hazardous conditions that are commonplace throughout mixed‐conifer forests of the Sierra Nevada and elsewhere in California (Safford & Stevens, 2017). The objectives of the original prescription in the FFS included mitigating hazards by emphasizing the mechanical removal of less fire‐resilient species and trees of smaller‐sized diameter classes (Figure 5A; Original). Prescribed fire greatly reduced surface fuels, yet the regular spacing in residual overstory from mechanical treatments in combination with prescribed fire stimulated moderate, yet uniform, levels of shrub cover. Additionally, prescribed fire following thinning killed residual trees from fire injury, which ultimately generated some CWD. Given our observations of how the effects of the FFS treatments changed over 20 years, our modified prescription for future treatments would include a hybrid of the Fire and Mech + Fire treatments (Figure 5A; Modified Future). The resulting stand would contain lower tree densities than our original prescription that are dispersed in irregularly spaced clumps. These conditions would be more in alignment with the resilient conditions seen historically, specifically a low competitive environment for overstory trees (North, Tompkins, et al., 2021), and greater heterogeneity in overstory tree arrangement (Lydersen et al., 2013). Our focus would be to increase the removal of less fire‐resistant species and promote more fire‐resistant species such as California black oak (*Quercus kelloggii* Newberry) and pines. By creating canopy gaps via mechanical treatments and repeated fire, we could promote the regeneration of desired species. Although increased light availability may also increase shrubs in the understory, repeated prescribed burns or mastication can maintain a desirable level of surface fuels. We may see increases in CWD following repeated fires due to fire‐caused injury or bark beetle infestation (Stark et al., 2013). However, the overall resilient conditions created by this modified treatment would still mitigate fire hazards and achieve the ecological benefits that result from active stewardship.

### Montana site

Prior to treatment, the Montana site was second‐growth forest with an overstory of 70–100‐year‐old ponderosa pine and Douglas‐fir (*Pseudotsuga menziesii* [Mirb.] Franco var. glauca [Beissn.] Franco), with a minor component of lodgepole pine (*Pinus contorta* var. *latifolia*) and western larch (Fiedler et al., 2010; Metlen & Fiedler, 2006). The historical fire return interval was 2 to 14 years (mean 7 years) (Grissino‐Mayer et al., 2006), but no fires had occurred in several decades. This long fire‐free interval had allowed a dense overstory and mid‐story of shade‐tolerant Douglas‐fir to develop and reach fire‐resistant sizes, with little ponderosa pine regeneration, creating conditions vulnerable to both high‐severity wildfire and bark beetles (Figure 5B; Control). Such conditions are common in the lower elevation forests of the Northern Rockies (Clyatt et al., 2016; Hood et al., 2021). The primary objectives for the Montana treatments were to reduce the likelihood of high‐severity wildfire and to restore the site to a forest structure and composition that more closely resembled historical reference conditions of ponderosa pine‐dominated, fire‐maintained forests (i.e., relatively open, spatially complex structure, dominated by large ponderosa pine). Thinning from below was prescribed to quickly restore forest structure and species composition by harvesting mostly Douglas‐fir to increase ponderosa pine dominance and encourage conditions conducive to ponderosa pine and western larch regeneration (see Fiedler et al., 2010 for prescription details). Prescribed burning after thinning was done to reduce seedling and small tree density and fuels generated from the harvest to extend the efficacy of the treatment and create more heterogenous conditions within each unit and to expose mineral soil to favor ponderosa pine and western larch germination (Figure 5B; Original). Four years post‐treatment, the site was affected by a regional mountain pine beetle (*Dendroctonus ponderosae* Hopkins) outbreak. Mountain pine beetle‐driven overstory pine mortality levels were high in the Control and Fire treatments over the course of the outbreak between 2005 and 2012, leading to similar live ponderosa pine basal area across all four treatments (Hood et al., 2016), but very different fuel loads and forest structure, by the end of the outbreak compared with the thinning and combined thinning and burning (Mech, Mech + Fire) treatments. In the control, the outbreak created numerous pine snags that have since fallen, increasing coarse woody fuel loads; existing small Douglas‐fir have increased in size and density, with limited ponderosa pine regeneration (see Hood et al., 2024 in this Special Feature). In contrast, very little pine mortality occurred during the outbreak in the Mech and Mech + Fire treatments. Conditions 20‐years post‐treatment remain open with clear separation between the overstory and understory fuels strata. Saplings are still mostly Douglas‐fir; however, ponderosa pine seedlings have greatly increased relative to Douglas‐fir over time and much more than in the control. Nevertheless, it is clear that the aggradation of surface and ladder fuels requires a follow‐up treatment for the stands to remain resistant to future disturbances such as wildfire and beetle outbreak. Given the ensuing effects of the mountain pine beetle outbreak and other observations over the 20 years since treatment, our modified treatment recommendation is to establish a cutting cycle of approximately 20–30 years, but to slightly reduce the basal area target relative to original prescription (10.3 m^2^ ha^−1^), incorporate more openings for structural complexity and age‐class diversity, and burn the site more frequently (Figure 5B; Modified Future). Prescribed burning every 7–20 years would likely encourage ponderosa pine and western larch regeneration over Douglas‐fir, but the burns will need to be patchy to not kill all previously established pine and larch. Vigorous and larger trees would be favored for retention during harvests. This modified prescription should create conditions resilient to high‐intensity wildfire, bark beetle outbreaks, and droughts.

### North Carolina site

Prior to treatment, the overstory of the North Carolina site was dominated by mature (~80‐year‐old) oaks, with a minor component of mature yellow pines (e.g., shortleaf pine [*P. echinata* Mill.] and pitch pine [*P. rigida* Mill.]) and hickories. These species accounted for approximately 75% of the total basal area. The remaining basal area consisted primarily of mesophytic hardwoods such as yellow‐poplar (*Liriodendron tulipifera* L.) and red maple (*Acer rubrum* L.), along with an ericaceous tree species (sourwood; *Oxydendrum arboreum* [L.] DC)—most of which had likely recruited into the overstory following several decades of fire exclusion. Mesophytic species dominated the regeneration layer, and ericaceous shrubs such as mountain laurel (*Kalmia latifolia* L.) formed a dense layer of ladder fuels throughout much of the study area. Woody fuel loads were substantial, and a thick layer of duff had also accumulated (Figure 5C; Control). Stand structure was changed by both the fire and combination treatments, with higher fire intensities in the latter resulting in the creation of some canopy gaps. Reductions in duff in these two treatments will likely reduce the risk and severity of a future wildfire. However, no treatment significantly altered overstory composition, and mesophytic and ericaceous species continue to recruit into the overstory (Figure 5C; Original). As these trees get larger, they become difficult to kill with fire without also killing oaks. Thus, a modified prescription that involves the selective harvesting of mesophytic trees—coupled with frequent burning (that may be refined to meet specific objectives by varying intensities and severities)—may be necessary. Care should be taken to ensure that the resultant canopy gaps are large enough to promote the recruitment of oaks, but not so large that light‐demanding trees like yellow‐poplar are able to outcompete them (Patterson et al., 2022). Additionally, an herbicide treatment targeting resprouting shrubs would effectively keep ladder fuels in check while further releasing oak seedlings and herbaceous groundcover from competition (Figure 5C; Modified Future).

## CONCLUSION

At year 20, the FFS study continues to provide new insights into the efficacy of fuel treatments across different regions in the United States. With this Special Feature, we were able to aggregate unique, long‐term datasets that encompassed a range of forest types, mechanical prescriptions, and prescribed fire implementation. Early evidence after the nationwide study ended in 2009 suggested that Mech + Fire maximized fuel reduction and ecological benefits in the short term. While this statement holds true ~20 years later, it must be contextualized by place: Mech + Fire produced more desirable outcomes in pine‐dominated forests in the long term while Fire only was a more effective treatment in hardwood‐dominated forests. Although each site was subjected to varying degrees of mechanical and prescribed fire treatments, the consensus across sites remains the same: fuel and restoration treatments work. Not only are they effective in the short term, but we can ensure that desired conditions, such as reduced fuel hazards and restoration, are maintained when followed up with frequent repeated treatments (as seen in California, Ohio, and North Carolina).

We recognize that implementation of restoration treatments at large spatial scales, let alone repeated or maintenance treatments, is difficult given constraints that limit the opportunities to do mechanical (North et al., 2015) or prescribed fire treatments (Schultz et al., 2019). Workforce capacity and regulatory hurdles (Clark et al., 2024) are additional challenges that further limit the scale of implementation. While we may never fully overcome these limitations, the results from this analysis of four very different forest types spanning most of the continental United States provide encouraging news to managers. These frequent fire‐adapted forests responded well to initial (Montana) and repeated treatments (California, Ohio, North Carolina) designed to reduce fire hazards and restore ecosystem processes. One key element of a successful program in forest restoration is the need for repeated treatments to maintain long‐term resilience. Future treatments can accommodate new goals as shown in our Modified Future treatments (Figure 5). Modified Future treatments included more spatial heterogeneity in forest structure and species shifts that should better accommodate the impacts of climate change. This highlights the critical need to keep the remaining FFS network operational along with other long‐term studies (Brodie et al., 2024; Radcliffe et al., 2024) that examine the effects of restoration and fuels reduction. Investing in such long‐term research is a priority, and it will require work to create longevity across institutions to keep the studies going as people retire or move to other locations.

## CONFLICT OF INTEREST STATEMENT

The authors declare no conflicts of interest.

## Supporting information


Appendix S1:

## Data Availability

Data (Bernal et al., 2024) are available on Dryad: https://doi.org/10.5061/dryad.5tb2rbpdp.
