# Supplementary material for: The national Fire and Fire Surrogate study: Effects of fuel treatments in the Western and Eastern United States after 20 years
Source: Ecol Appl. 2025 Feb 12;35(1):e70003. doi: 10.1002/eap.70003 (PMC11817480; doi:10.1002/eap.70003)
Supplement: Supplementary file 1 — Appendix S1: [file EAP-35-e70003-s001.pdf]

The national Fire and Fire Surrogate study: Effects of fuel treatments in the western and eastern United States after 20 years

Alexis A. Bernal, Scott L. Stephens, Mac A. Callaham, Brandon M. Collins, Justin S. Crotteau, Matthew B. Dickinson, Donald L. Hagan, Rachelle Hedges, Sharon M. Hood, Todd F. Hutchinson, Melanie K. Taylor, and T. Adam Coates

Ecological Applications

Appendix S1

Table S1 Definitions of terms used to describe details of mechanical and prescribed fire treatments.

Term	Definition
Mechanically thinned	Use of mechanized equipment to remove biomass
Hand thinned	Use of hand tools to remove biomass
Thinned from below	Removal of biomass that generally consists of the mid- to understory vegetation
Mastication	Fuels treatment that chips/grinds biomass and leaves that biomass on site
Salvage harvest	Removal of biomass (often with merchantable value) after tree mortality occurs
Broadcast burn	Fire applied across a pre-determined area using various ignition patterns
Strip head fire	Ignition pattern used in a broadcast burn that is initiated at the bottom of the slope in a linear pattern (i.e., strips) and burns upward and perpendicular to the slope
Spot fire	Ignition pattern used in a broadcast burn that uses spots of fire instead of strips.

Helicopter ignition	Ignitions from a helicopter to initiate a broadcast burn
Hand ignition	Ignitions using hand tools (e.g., drip torches) to initiate a broadcast burn

Table S2 Summary of canonical discriminant performance metrics across each site including (from left to right) correlation across variable for canonical axis 1 and 2 (p-value), scores for axis 1 and 2 in each treatment, and p-values from testing differences across treatments before and ~20-years after treatment using multiresponse permutation procedure (MRPP). Values in bold indicate significance based on α = 0.05.

Site			Treatment Scores (Can1, Can2)				MRPP	
Overstory	Can1	Can2	Control	Fire	Mech	Mech+Fire	Pre-treatment	Post-20
California	**85 (<0.01)**	**8 (<0.01)**	1.88, -0.27	0.27, 0.27	-0.74, 0.49	-1.61, 0.52	0.65	**0.02**
Montana	**71 (<0.01)**	**21 (<0.01)**	1.65, 0.57	0.86, -0.97	-0.87, 0.74	-1.56, -0.35	0.98	**0.01**
Ohio	**67 (<0.01)**	**26 (<0.01)**	-1.24, -0.78	0.87, -0.53	-0.84, 0.99	1.35, 0.33	0.92	**0.01**
North Carolina	**79 (<0.01)**	**13 (0.03)**	-1.11, -0.48	-0.08, 0.02	-0.39, 0.64	1.58, 0.18	0.45	**<0.01**
Understory								
California	**57 (<0.01)**	**28 (0.01)**	-0.26, -0.22	0.36, 0.37	-0.55, -0.14	0.42, 0.33	0.99	0.52
Montana	**78 (0.01)**	16 (0.48)	0.51, 0.14	0.65, -0.18	-0.50, 0.39	-0.71, -0.31	0.16	0.99
Ohio	**98 (<0.01)**	1 (0.72)	-1.99, 0.18	1.48, 0.27	-1.56, -0.24	2.07, -0.20	0.37	**0.01**

North	**82**	**14**	-1.18,	0.20,	-0.41,	1.38,	0.43	**0.01**
Carolina	**(<0.01)**	**(0.04)**	0.38	0.14	-0.66	0.12		
Fuels								
California	**82**	**17**	-1.06,	0.47,	-0.34,	0.90,	0.99	**<0.01**
	**(<0.01)**	**(<0.01)**	0.30	0.20	-0.59	0.02		
Montana	**73**	25	-0.75,	-0.33,	0.53,	0.59,	0.36	**0.04**
	**(<0.01)**	(0.11)	-0.20	0.32	-0.46	0.31		
North	**92**	7	-0.85,	0.28,	-0.42,	0.98,	0.97	0.22
Carolina	**(<0.01)**	(0.23)	0.22	0.04	-0.30	0.05		
